# The Dispensatory of the United States

**Published:** 1858-05

**Authors:** 


ART. IV.—*The Dispensatory of the United States of America.* By GEORGE B. WOOD, M.D., and FRANKLIN BACHE, M.D. Eleventh edition; carefully revised. Philadelphia: J. B. Lippincott & Co. 8vo. pp. 1583. 1858.

WE have great pleasure in announcing a new edition, the eleventh, of this world-wide renowned work since its first appearance in 1833. To say anything in commendation of it, at this late period, would be entirely superfluous, since it is to be found in the library of every intelligent physician in the country. In preparing the present edition, the learned authors have spared no pains to add to it every discovery and improvement of any value in materia medica and pharmacy. Their object has been, as they have stated in the preface, to make the work a mirror reflecting the existing state of these two sciences; and no one acquainted with their labor can doubt the success of the enterprise. Large additions have been made to materia medica and pharmacy within the last few years, and the reader can form some idea of the labor expended in the preparation of the work, when he is informed that it contains one hundred pages of new matter.

